# COVID-19 Morbidity and Mortality in Social Networks: Does It Influence Vaccine Hesitancy?

**DOI:** 10.3390/ijerph18189448

**Published:** 2021-09-07

**Authors:** Jagdish Khubchandani, Sushil Sharma, James H. Price, Michael J. Wiblishauser, Fern J. Webb

**Affiliations:** 1 Department of Public Health Sciences, New Mexico State University, Las Cruces, NM 88003, USA; 2 Miller College of Business, Ball State University, Muncie, IN 47306, USA; ssharma@bsu.edu; 3 School of Population Health, University of Toledo, Toledo, OH 43606, USA; jprice@utnet.utoledo.edu; 4 School of Education, Health Professions & Human Development, University of Houston, Victoria, TX 77901, USA; wiblishauserm@uhv.edu; 5 Department of Community Health and Family Medicine, University of Florida, Jacksonville, FL 32209, USA; Fern.Webb@jax.ufl.edu

**Keywords:** COVID-19, Coronavirus, vaccine, hesitancy, denial, behavior, immunization

## Abstract

The impact of COVID-19 morbidity and mortality among family and friends on vaccination preferences is not well explored. A valid and reliable questionnaire was deployed online via mTurk to recruit a national random sample of adult Americans to understand COVID-19 vaccination preferences and its relationship with COVID-19 infection in social networks. A total of 1602 individuals participated in the study where the majority had taken at least one dose of the COVID-19 vaccine (79%) and almost a tenth were planning to do so (10%) or did not want to take the vaccine (11%). Compared to those who knew family members or friends affected by COVID-19, those who did not know anyone infected with (AOR = 3.20), hospitalized for (AOR = 3.60), or died of COVID-19 (AOR = 2.97) had statistically significantly higher odds of refusing the vaccines. Most strategies for reducing COVID-19 vaccination hesitancy focus on highlighting the benefits of COVID-19 vaccines. We suggest that the dangers of not getting the vaccine should also be emphasized as many people who do not know someone who was affected with COVID-19 are also hesitant towards vaccination. These individuals may not fully appreciate the morbidity and mortality impact of COVID-19 infections and the messaging can be tailored to highlight the risk of not having vaccines.

## 1. Introduction

Mass vaccination campaigns for COVID-19 started worldwide in early 2021. Amidst the many challenges with COVID-19 vaccination campaigns around the world, vaccine hesitancy received substantial attention from the popular press and in the scientific literature [1,2,3]. However, most reports on COVID-19 vaccine hesitancy focus on the sociodemographic characteristics of individuals. For example, multiple national studies and systematic reviews have linked COVID-19 vaccine hesitancy to differences in age, sex, education, marital status, race, and occupation of study participants [1,2,3,4]. The few studies that focus on cognitive correlates have assessed differences among individuals’ behaviors, knowledge, and perceptions as it relates to COVID-19 vaccination hesitancy. Many of these studies indicate that individuals with more knowledge and greater perceived susceptibility to COVID-19 infections are more likely to accept vaccinations [3,4,5,6]. However, little is known about the impact of COVID-19 morbidity and mortality among family and friends on vaccination preferences. To address this, we conducted a national assessment with adult Americans to understand how COVID-19 infections in social networks can influence COVID-19 vaccination willingness.

## 2. Methods

A multi-item valid and reliable questionnaire was deployed via Amazon mTurk in June 2021 across the United States after approval from the Institutional Review Board. To ensure face validity, the survey was developed based on a comprehensive review of the literature and existing surveys on COVID-19 vaccination hesitancy [1,2,3,6]. Subsequently, the survey was reviewed by a panel of experts (*n* = 3) to ensure content validity, and based on expert feedback, the questionnaire was modified. First, we asked participants if they had received a COVID-19 vaccine (with response options: yes, one dose; yes, both the doses; not received the vaccine, but plan to get one; and I do not want the vaccine). Second, we asked study participants do “you personally know someone (i.e., family members or friends) who tested positive for COVID-19 infection, were hospitalized due to COVID-19 or died due to COVID-19 infection” (with response options: yes, one person; yes, more than one person; no, I do not know anyone who has been infected with, hospitalized for, or has died due to COVID-19). We computed the internal consistency reliability coefficient for these three COVID-19 in social networks questions, which was found to be high (Cronbach alpha = 0.79). Finally, we asked the study participants about sociodemographic characteristics that have been associated with COVID-19 vaccination willingness in previous reports and across multiple studies. To ensure external validity, a power analysis was conducted for sample size estimation. Based on the total approximate population of adults in the USA (*n* = 50 million), 99% confidence levels, and a conservative 3% margin of error, a total of 1383 participants were needed for the study [3,7].

## 3. Results

A total of 1602 individuals participated in the study with the majority (>50%) being male, White, non-Hispanic, 18–35 years old, married, urban, employed full time, and with a college degree (Table 1). Vaccination willingness was distributed as: taken at least one dose of the vaccine (79%), not taken the vaccine, but planning to do so (10%), and do not want to take the vaccine (11%). Vaccine refusal was statistically significantly higher among non-Hispanic Blacks, suburban dwellers, those with less than a college education, non-married individuals, and political conservatives or independent leaning participants. Individuals who did not know anyone among family or friends infected with, hospitalized for, or who died of COVID-19 were significantly more likely to refuse the vaccines (Table 1).

Logistic regression analyses were conducted to assess the association between COVID-19 vaccine refusal and having someone in social networks who was infected, hospitalized, or who died due to COVID-19 (Table 2). In the unadjusted analysis (model 1), vaccine refusal was significantly higher among those who did not know anyone infected with, hospitalized for, or who died of COVID-19 compared to individuals who knew someone affected by COVID-19 among social network members. Despite adjusting for all the sociodemographic characteristics (Table 1) that could lead to differences in vaccine willingness, those without exposure to COVID-19 infections among social networks were more likely to refuse the vaccines (model 2). Compared to those who knew family members or friends affected by COVID-19, those who did not know anyone infected with (AOR = 3.20), hospitalized for (AOR = 3.60), or who died of COVID-19 (AOR = 2.97) had statistically significantly higher odds of refusing the vaccines (Table 2). In all the adjusted regression models, education and political affiliation were also significant predictors of vaccine refusal (in addition to COVID-19 infection, hospitalization, and deaths among family members and friends).

## 4. Discussion

Several studies from the general adult population have delineated the sociodemographic predictors of COVID-19 vaccination hesitancy in the United States. These predictors include race, gender, age, region of residence, income, family structure, employment status, education, and political affiliation [2,3,4]. In this large national study of adult Americans, similar to earlier studies, we found that education and political affiliation were associated with COVID-19 vaccination hesitancy. Additionally, we found strong associations between COVID-19 vaccine willingness and whether or not family members or friends were afflicted with COVID-19. Earlier studies have shown that individuals with greater perceived risk and fear of COVID-19 were more willing to take the vaccines [1,3]. It could be possible that knowing someone affected with COVID-19 could increase the perceived risk and fear of COVID-19 infection, which in turn could increase willingness for COVID-19 vaccination. If so, this is a novel finding of our study with critical implications for reducing COVID-19 vaccination hesitancy among the general public. Most recommendations and strategies for reducing COVID-19 vaccination hesitancy focus on highlighting the benefits of COVID-19 vaccines and providing information to the general public about the vaccines [4,5,6,8,9,10]. In addition, a widely acknowledged strategy to reduce COVID-19 vaccine hesitancy emphasizes the 5Cs (reduce Complacency and Constraints, increase Confidence and Calculations in favor of vaccines, and promote Collective responsibility) [8,9,10]. We suggest the addition of more Cs to initiatives geared at increasing COVID-19 vaccine willingness (i.e., Communication for a unique local Context with Comparative analyses of the risks of having COVID-19 infection versus COVID-19 vaccination). Such communication, along with addressing public concerns (e.g., of safety and side effects of vaccines) could be especially useful among those who do not have favorable attitudes towards vaccinations [8,9,10].

Early in the pandemic, it was believed that COVID-19 infections predominantly affected the elderly and sicker individuals. Moreover, many younger individuals had a lower perceived risk of severe outcomes from COVID-19. To counter such misbeliefs and based on the results of this study, clinicians in healthcare facilities and community advocates for vaccinations have opportunities to raise awareness and educate individuals about the severity and extent of COVID-19 infection-related morbidity and mortality, along with the benefits of vaccines [9,10,11]. For example, more than 40 million Americans have been infected with COVID-19, and out of these, more than 650,000 have died. In contrast, more than half of adult Americans are now fully vaccinated for COVID-19 with a very small amount of breakthrough infections and deaths seen among vaccinated individuals [11,12]. Our analysis suggests a novel addition to the existing tools and interventions to encourage the uptake of COVID-19 vaccines by emphasizing the benefits of vaccines along with educating the public on the risk of acquiring infections and suffering serious consequences from infections in the absence of vaccination.

The results of this study must be viewed in light of potential limitations. The results of the study are restricted by all the threats to the validity and reliability inherent to survey study designs (e.g., reliance on self-reported behaviors, recall bias in participants, socially desirable responses, and the inability to establish cause-and-effect relationships) [3,7]. Furthermore, a major threat to the external validity is that the sample is limited in nature (e.g., limited to those with computers and understanding of the online survey environment or those with higher education). The COVID-19 vaccination rates observed in this sample (79%) were higher than the average for adults in the US (60%) when the data were collected [11,12]. Additional studies are required with larger samples of unvaccinated individuals to further understand the interplay of the sociodemographic and cognitive factors associated with COVID-19 vaccine hesitancy. Despite the limitations, this is one of the largest national studies that explore the influence of COVID-19 infection in social networks on vaccine willingness. Clinicians, policymakers, and public health practitioners should utilize a variety of messaging strategies to increase vaccination uptake and continue to monitor COVID-19 vaccination trends and preferences.

## Figures and Tables

**Table 1:** Demographic Characteristics, COVID-19 Infection in Social Networks, and Vaccine Willingness *.

Variable		Total N (%)	Taken at Least One Dose of the Vaccine	Not Taken, but Plan to Take the Vaccine	Do Not Want to Take the Vaccine	*p* Value
**All Participants**		1602 (100)	1277 (79)	159 (10)	166 (11)	
**Sex**	Male	1034 (65)	830 (80)	95 (9)	109 (11)	0.28
	Female	552 (35)	435 (79)	64 (11)	53 (10)	
**Race**	White	1293 (81)	1040 (80)	134 (10)	119 (9)	0.007
	African-American	161 (10)	121 (75)	11 (7)	29 (18)	
	Asian	86 (5)	69 (80)	10 (12)	7 (8)	
	Other	62 (4)	47 (76)	4 (7)	11 (18)	
**Ethnicity**	Hispanic	459 (29)	415 (90)	18 (4)	26 (6)	0.01
	Non-Hispanic	1127 (70)	850 (75)	141 (13)	136 (12)	
**Age Group**	18–25 years	199 (12)	162 (81)	23 (12)	14 (7)	0.51
	26–35 years	704 (44)	558 (79)	67 (10)	79 (11)	
	36–45 years	343 (21)	282 (82)	30 (9)	31 (9)	
	46–59 years	243 (15)	185 (76)	29 (12)	29 (12)	
	≥60 years	95 (6)	77 (81)	10 (11)	8 (8)	
**Marital Status**	Single/never married	317 (20)	212 (67)	52 (16)	53 (17)	<0.001
	Married	1174 (73)	993 (85)	89 (8)	92 (7)	
	Engaged/living with a partner	46 (3)	25 (54)	11 (24)	10 (22)	
	Divorced/separated/widow	49 (3)	35 (71)	7 (14)	7 (15)	
**Education**	High school or less	90 (6)	44 (49)	12 (13)	34 (38)	<0.001
	Some college experience	149 (9)	93 (62)	27 (18)	29 (20)	
	Bachelor’s degree	992 (62)	822 (83)	96 (10)	74 (8)	
	≥Master’s degree	355 (22)	306 (86)	24 (7)	25 (7)	
**Current Employment Status**	Full-time	1393 (87)	1121 (81)	137 (10)	135 (9)	0.12
	Part-time	116 (7)	91 (78)	10 (9)	15 (13)	
	Not employed	77 (5)	53 (69)	12 (16)	12 (16)	
**Area of Residence**	Rural	495 (31)	428 (86)	34 (7)	33 (7)	0.01
	Urban	802 (50)	635 (79)	96 (12)	71 (9)	
	Suburban	289 (18)	202 (70)	29 (10)	58 (20)	
**Political Affiliation**	Democrat	968 (61)	802 (83)	98 (10)	68 (7)	0.01
	Republican	426 (27)	340 (80)	33 (8)	53 (12)	
	Independent	172 (11)	113 (60)	24 (14)	35 (20)	
	Other	20 (1)	10 (50)	4 (20)	6 (30	
**COVID-19 infection among family/friends**	Yes, one person	645 (40)	574 (89)	31 (5)	40 (6)	<0.001
	Yes, more than one person	659 (41)	532 (81)	72 (11)	55 (8)	
	No one in family or friends had COVID-19	298 (19)	171 (57)	56 (19)	71 (24)	
**COVID-19 hospitalization of family/friends**	Yes, one person	704 (44)	630 (90)	39 (6)	35 (5)	<0.001
	Yes, more than one person	481 (31)	406 (84)	43 (9)	32 (7)	
	No one in family or friends hospitalized	417 (26)	241 (58)	77 (19)	99 (24)	
**COVID-19 related death in family/friends**	Yes, one person	635 (40)	573 (90)	31 (5)	31 (5)	<0.001
	Yes, more than one person	358 (22)	308 (86)	31 (9)	19 (5)	
	No one in family or friends died of COVID	609 (38)	396 (65)	97 (16)	116 (19)	

N (%) indicates the number of participants in each response category and percentages. the *p*-value indicates statistical significance for group differences in Chi-square tests; * % may vary due to rounding or missing values.

**Table 2:** Regression Analyses to Predict COVID-19 Vaccination Refusal Based on COVID-19 Exposure in Social Networks.

COVID-19 Morbidity and Mortality in Social Networks		Probability of Vaccine Refusal	
		Model 1 OR (95% CI)	Model 2 AOR (95% CI)
COVID-19 infection among family/friends	Yes	Ref 3.98 (2.84–5.59) *	Ref 3.20 (2.21–4.61) *
	No		
COVID-19 related hospitalizations among family/friends	Yes	Ref 5.20 (3.72–7.26) *	Ref 3.60 (2.48–5.21) *
	No		
COVID-19 related deaths among family members/friends	Yes	Ref 4.43 (3.13–6.30) *	Ref 2.97 (2.03–4.36) *
	No		

* indicates *p* < 0.05. OR = odds ratios, AOR = adjusted odds ratios, 95% CI = confidence intervals. The binary outcomes were vaccine refusal vs. taken the vaccine or planning to take the vaccine. Ref indicates reference group for comparison with those who did not have COVID-19 related morbidity or mortality in social networks. Model 1 shows unadjusted regression analysis to predict vaccine refusal based on COVID-19 exposure in social networks. Model 2 shows multiple regression analysis to predict vaccine refusal after adjusting for all the sociodemographic characteristics from Table 1. In model 2, education & political affiliation were statistically significant moderators of the relationship between vaccine refusal and COVID-19 in social networks.

## Data Availability

The data presented in this study are available on request from the corresponding author.
